# Affinity-Based Analysis Methods for the Detection of Aminoglycoside Antibiotic Residues in Animal-Derived Foods: A Review

**DOI:** 10.3390/foods12081587

**Published:** 2023-04-08

**Authors:** Zhaozhou Li, Yanyan Liu, Xiujin Chen, Yao Wang, Huawei Niu, Fang Li, Hongli Gao, Huichun Yu, Yunxia Yuan, Yong Yin, Daomin Li

**Affiliations:** Henan International Joint Laboratory of Food Green Processing and Quality Safety Control, College of Food and Bioengineering, Henan University of Science and Technology, Luoyang 471000, China; ilizhaozhou@126.com (Z.L.); 18436089312@163.com (Y.L.);

**Keywords:** aminoglycosides antibiotics residues, immunological detection methods, animal-derived foods, food safety inspection

## Abstract

With the increasingly serious problem of aminoglycoside antibiotic residues, it is imperative to develop rapid, sensitive and efficient detection methods. This article reviews the detection methods of aminoglycoside antibiotics in animal-derived foods, including enzyme-linked immunosorbent assay, fluorescent immunoassay, chemical immunoassay, affinity sensing assay, lateral flow immunochromatography and molecular imprinted immunoassay. After evaluating the performance of these methods, the advantages and disadvantages were analyzed and compared. Furthermore, development prospects and research trends were proposed and summarized. This review can serve as a basis for further research and provide helpful references and new insights for the analysis of aminoglycoside residues. Accordingly, the in-depth investigation and analysis will certainly make great contributions to food safety, public hygiene and human health.

## 1. Introduction

### 1.1. Structures and Properties of Aminoglycoside Antibiotics

**Figure 1:**
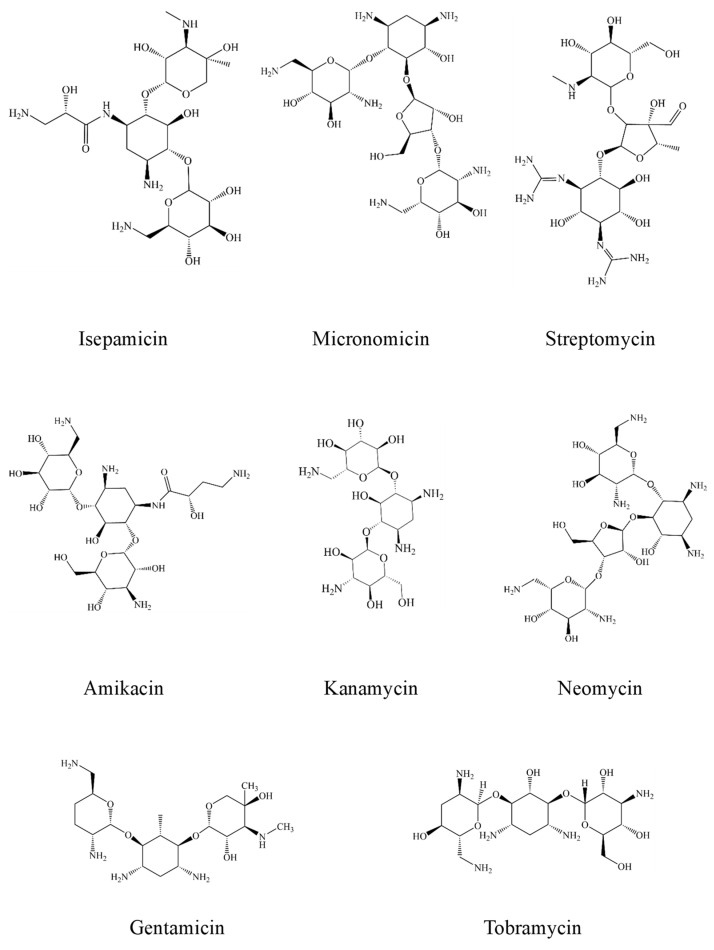
Chemical structure diagram of aminoglycoside antibiotics.

Aminoglycoside antibiotics (AGs) are highly potent broad-spectrum antibiotics, whose molecules are compounded and connected by an amino sugar and aminocyclitol alcohol through an oxygen bridge [1,2]. The chemical structures are shown in Figure 1. AGs are mainly divided into natural and amino-modified semi-synthetic glycosides. Their molecular structure consists of 2-deoxystreptamine (2-DOS) as the central unit, linked with 2–3 sugar units by glycosidic bonds [3]. Moreover, AGs are highly polar and hydrophilic with an oil-water partition coefficient in the range of −4 to −9. They are extremely soluble in water, slightly soluble in methanol and insoluble in non-polar organic solvents [4]. AGs mainly include Streptomycin (STR), Dihydrostreptomycin (DHS), Kanamycin (KAN), Neomycin (NEO), Tobramycin (TOB), Gentamicin (GEN), Spectinomycin (SPE), Amikacin (AMK) and so on [5] (Figure 1). As an important antimicrobial agent, AGs can kill bacteria during the rest period by inhibiting the synthesis of bacterial proteins and disrupting the integrity of cell membranes [6,7]. Due to their broad-spectrum antimicrobial activities against gram-positive and gram-negative bacteria, they are widely used to treat bacterial infections in animals and humans and promote animal growth by improving feed efficiency [8,9,10].

### 1.2. Hazards of Aminoglycoside Antibiotics

With the excessive, irregular and irrational utilization of AGs, the resulting residues are usually found in milk [11,12], honey [1,13,14,15,16], meat [17,18] and other foods, endangering food safety, environmental damage and human health hazards [19,20]. AGs residues can result in a number of toxicities, including nephrotoxicity, ototoxicity, hematopoietic toxicity, damage to the vestibular nerve, muscle paralysis and allergy [21,22,23,24,25,26]. Common allergic reactions can be confirmedly diagnosed as rash and urticaria [27]. The symptoms of neuromuscular blockade include myocardial depression, decreased blood pressure, tingling and numbness in the hands and feet [28]. Ototoxicity includes vestibular dysfunction manifest as dizziness, nausea and vomiting. Cochlear nerve damage can cause permanent hearing loss [29]. In severe cases, shock and even death may occur, causing considerable harm to human health [30]. Furthermore, AGs are highly toxic in nature and are transmitted to the human body through the food chain, resulting in significant health risks [31]. Additionally, AGs residues in foods of animal origin can lead to antibiotic resistance in human body, which represents a serious health hazard to human beings.

### 1.3. Maximum Residue Limits of Aminoglycoside Antibiotics

With the rapidly growing misuse and abuse of AGs, the adverse reactions including allergies, organ damage and bacterial drug resistance caused by them have been attracting a great deal of attention. Therefore, the European Union, the United States, Japan and China have established the corresponding maximum residual limits for AGs in animal-derived foods [26,32,33,34,35] (Table 1). For example, both the European Union and China have a limit of 100 µg/kg for KAN residues in muscle and fat of swine and cattle, Japan set a standard of 40 µg/kg [36,37]. In the United States, the residual limit of NEO in the muscle was defined as 1200 μg/kg in pork [38].In the European Union, the residue limits of DHS in the muscle and liver were 500 µg/kg in sheep. Additionally, the STR residual limit was established as 600 μg/kg in chicken edible tissues in China. In the European Union and Japan, the limits for KAN residues in the muscle and kidney of swine were all lower than NEO. In China, the residual limit of DHS in the muscle of cattle was higher than GEN. Additionally, STR had the same residue limit as DHS in edible tissues of sheep in the European Union, China and Japan.

## 2. Simple Sample Pretreatment Methods

AGs widely exist in different sample matrices. According to the characteristics of the matrices and the requirements of detection methods, sample extraction methods should be compared and optimized to further meet the requirements of AGs residue analysis [26]. At present, for extraction and purification of trace AGs, commonly used pretreatment methods include solid phase extraction (SPE) [39,40,41], molecular imprinting solid phase extraction (MISPE) [42], liquid-liquid extraction (LLE) [17,43], pressurized liquid extraction (PLE) [44], matrix solid phase dispersion (MSPD) [45], dilute ultrasound-assisted extraction, membrane filtration and so on. AGs sample pretreatment methods are shown in Table 2. It can be seen from the table that the sample pretreatment of AGs in animal foods such as milk, honey, eggs, milk powder and meat can reduce the interferences of complex matrices and improve the signal intensities of the target analytes. In addition, sample pretreatment concentrates on trace residues, improves the sensitivity and selectivity of the detection performance, making them suitable for further residue analysis.

## 3. Non-Targeting Detection Methods

Residue analysis of AGs is an important measure to ensure food safety and human health. Traditional detection methods are based on microbial methods [55]. The microbial detection methods can determine the residues of antibiotics qualitatively or semi-quantitatively according to the inhibitory effects of antibiotics on specific microorganisms [56]. However, these methods can only estimate biological activities, which are time consuming and the results are influenced by many factors, that may lead to poor sensitivity and specificity [57]. Alternatively, high-performance liquid chromatography (HPLC), gas chromatography (GC), liquid chromatography, liquid chromatography-tandem mass spectrometry (LC-MS/MS) and capillary electrophoresis (CE) have also been applied to the detection of AGs [2,8,40,58,59,60,61,62,63]. HPLC and LC-MS/MS are the most commonly used analytical methods. Since AGs have no chromophore, instrumental detection needs to be coupled with a pulsed amperometric detector (PAD), mass spectrometry (MS) and evaporative light-scattering detection (ELSD) [64]. Instrumental analysis has high sensitivity and accuracy, but it is expensive and is difficult for rapid on-site detection. These methods are all unfavorable to the rapid, selective, sensitive and high-throughput screening of AGs residues in the foods of animal origin.

## 4. Affinity-Based Analysis Methods for Aminoglycoside Residues

With the diversified development of science and technology and the cross-integration between different disciplines, the range of affinity-based detection technology has been expanding gradually and the research on related topics has been opening on an endless stream. Affinity-based analysis methods are established based on the specific recognition of metal ions, aptamers, antibodies and molecularly imprinted polymers [5,65,66,67]. These mainly include enzyme immunoassay [68,69,70,71,72,73], colloidal gold immunochromatography [74], aptamer sensing detection, lateral flow immunoassay [75], fluorescence immunoassay [76], chemiluminescence immunoassay [77,78,79] and so on. They have strong selectivity, high sensitivity, simple operation and low cost. At present, they are ideal methods for AGs residues screening. Furthermore, these methods reduce the requirements for operators, shorten the detection time and provide technical tools for rapid and efficient screening AGs residues in real samples. The detailed advantages and disadvantages of the affinity-based analysis methods were compared and summarized in Table 3. 

### 4.1. Enzyme-Linked Immunosorbent Assay

Enzyme-linked immunosorbent assay (ELISA) is a kind of immunoassay technology developed based on immunoenzymatic techniques. This method adsorbs soluble antigens/antibodies on the surface of solid-phase carriers and then integrates the specific recognition between antigens and antibodies with the highly catalytic effects of enzymes on the substrate. The sodium periodate method, mixed anhydride method, glutaraldehyde method and 1-ethyl-3-(3-dimethylaminopropyl) carbodiimide (EDC) method are usually used for enzyme labeling. Furthermore, O-phenylenediamine, tetramethyl benzidine (TMB) and 2,2’-Azinobis-(3-ethylbenzthiazoline-6-sulphonate) (ABTS) are often used as the catalytic substrates in the immunoassay. Due to its fast, sensitive, simple and high-throughput detection procedure [80], it is currently the most time-saving and widely used immunological method.

#### 4.1.1. Construction of Detection Models

Depending on the antigen fixation strategy, antibody labeling strategy and antibody-antigen reaction types (direct recognition or competition), the ELISA methods can be defined as direct, indirect, competitive and sandwich detection models. The competitive type can be divided into direct competitive enzyme-linked immunosorbent assay (dc-ELISA) and indirect competitive enzyme-linked immunosorbent assay (ic-ELISA).

Different detection models also have a certain impact on the detection results and performance. Jin et al. determined NEO residues with a detection limit (LOD) of 2.73 ng/mL by the dc-ELISA method in milk samples [81]. Although it does not require a lot of operators and instruments, the false negative results of this method are considerably high. Furthermore, the LOD of NEO was 0.08 ng/mL by the high-performance ic-ELISA method established by Xu et al. [72]. They used the ic-ELISA method to avoid the high false negative results and improved the sensitivity. In addition, Wang et al. used the ic-ELISA method to detect STR residues in animal-derived foods. That the half-maximum inhibitory concentration (IC_50_) of STR was 2.00 ng/mL, the LOD was 0.24 ng/mL and the sample recovery rate was from 71.32% to 106.94% [82]. This method is fast, simple, sensitive and suitable for the accurate screening of STR residues in foods of animal origin. Studies have shown that ic-ELISA is more sensitive than dc-ELISA. This is possible due to the fact that the binding rate of antigen to ic-ELISA is higher than solid phase antigens, but the labeled antigen and the antigen to be tested are the same as the binding rate to solid phase antigens in the dc-ELISA.

To improve the efficiency of detection methods, some reports have been implemented with multiplexing strategies, where multiple analytes are detected simultaneously with a single analysis procedure. Wei et al. established a two-point ELISA for the simultaneous detection of KAN and STR in milk. In the visual inspection model, the LODs were 2.7 ng/mL and 12.5 ng/mL, respectively. The quantitative detection ranges were calculated as 0.38 ng/mL–38.66 ng/mL and 2.47 ng/mL–31.90 ng/mL, respectively. The recovery rates were determined as 84.2–119.8% and 93.3–124.5%, respectively [70]. This two-point enzyme-linked immunoassay provides a new platform for multi-component analysis in a single model, enabling efficient monitoring of food quality and safety.

#### 4.1.2. Signal Enhancement Strategies

The marker synthesis, enzyme catalytic efficiency and recognition mechanism have been proved to play crucial roles in the ELISA detection. Abuknesha et al. prepared the artificial antigen by coupling STR, melamine and bovine serum albumin with a two-step method for the detection of STR in water samples, milk and serum samples. The linear range of the standard curve was 1 ng/mL–200 ng/mL and the LOD was determined as 1 ng/mL [83]. To further explore the synthesis method of antigens and broaden the application scope of ELISA, Chen et al. prepared an immunogen by the carbodiimide method and coupled with the carried protein by the glutaraldehyde method. The IC_50_ of GEN was 0.92 ng/mL. The LODs were 6.2 μg/kg for swine muscle, 3.6 μg/kg for swine liver and 2.7 μg/kg for swine kidney, respectively [84]. It is proved that the preparation method of antigen has a great influence on the performance of immunoassay. Therefore, improving and enhancing the ELISA detection performance can enable the better monitoring of the AGs residues.

**Figure 2:**
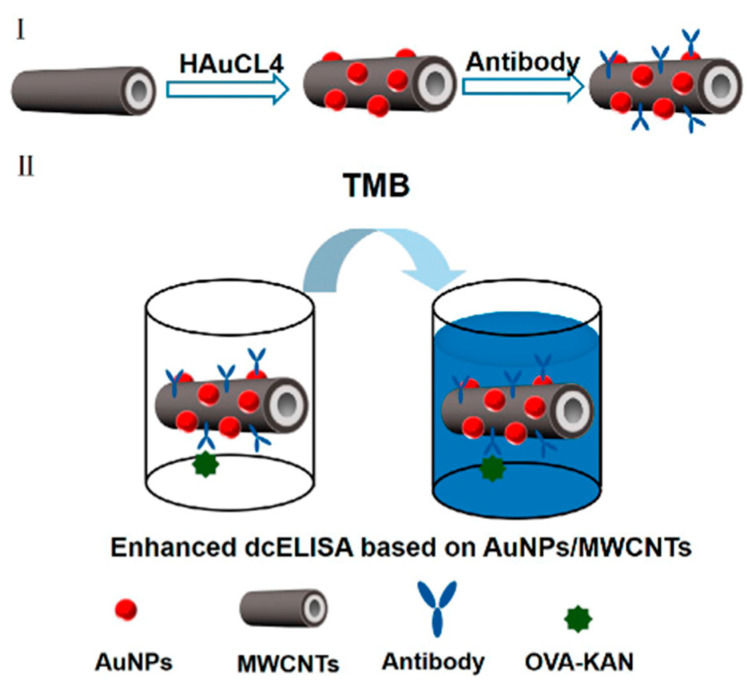
Schematic diagram of direct competitive enzyme-linked immunosorbent assay (dc-ELISA) based on Au/multiwalled carbon nanotubes (MWCNT). (**I**) Preparation of antibody labeled AuNPs/ MWCNTs nanohybrids; (**II**) Development of enhanced dcELISA based on AuNPs/MWCNTs [86] (reproduced with permission from John Wiley and Sons, Inc.).

Based on the traditional marker synthesis, the dc-ELISA method was further improved by introducing nanomaterials. Jiang et al. developed a novel HRP-Kana probe based on AuNPs modification and then established a dc-ELISA method with the hybridoma technology for the detection of KAN and TOB residues in milk [85]. Compared with the traditional dc-ELISA method (0.13 ng/mL), the LOD (0.022 ng/mL) was increased by 5 times. The result showed that the sensitivity can be highly improved with the AuNPs. At the same time, it laid a certain foundation for the trace residue detection in the complex matrix. To further explore the influence of nanomaterials on this detection method, Zeng et al. established a dc-ELISA method based on Au/multiwalled carbon nanotubes (MWCNT) nanohybridization for KAN detection in milk samples (Figure 2). Under the optimal conditions, the LOD was 11.2 pg/mL, the recovery rate was in the range of 94.3–124.5% [86]. This immunoassay demonstrated high precision and accuracy in real samples and improved the LOD of AGs residues from nanogram level to picogram level. It has great potential application prospects in food monitoring and quality control.

ELISA has been widely used in foods and has been the mainstream technology for rapid detection of food safety. Using this technique to analyze drug residues in foods has gradually become a new hotspot in academic research at home and abroad. However, the development of this method inevitably has possessed some drawbacks, such as a complicated antibody preparation process, high cost, poor stability, high cross reaction, low signal amplification and so on. This will inevitably lead to poor selectivity of the determination [68]. Furthermore, there are also shortcomings such as poor repeatability and high false negative results. To avoid or solve these problems, highly specific antibodies are essentially required. Therefore, it is necessary to develop and innovate the ELISA detection method and then develop a solid technical platform for antibiotic residues. Table 4 summarizes the applications of ELISA detection of AGs residues in the animal-derived foods. It can be concluded from the table that the ELISA method can be used to effectively monitor the residues and meet the requirements of MRLs in the detection of AGs in milk, chicken, pork, eggs and other animal-derived foods. In addition, recognition components and new signal tags can provide new ideas for expanding the application range of this method in the foods of animal origin.

### 4.2. Colloidal Gold Immunochromatographic Assay

Colloidal gold, also known as a gold solution, is a suspension of gold particles formed after the reduction in gold salts. Colloidal gold immunochromatographic assay (GICA) is a kind of immobile labeling immunoassay with colloidal gold as the marker and nitrocellulose film as the solid phase support. It integrates various analytical methods involving colloidal gold labeling technology, immunodetection technology and protein chromatography technology with specific reactions between antigens and antibodies. This method can achieve a qualitative or semi-quantitative analysis of the target compounds.

GICA has been widely used in AGs detection due to its advantages of a wide application range, stable marker and low cost. Jin et al. conjugated neomycin, KAN and GEN with the carrier proteins as the artificial antigens to prepare monoclonal antibodies for the AGs detection. The results showed that the LODs were 10 ng/mL, 8 ng/mL and 6 ng/mL in PBS, plasma and milk samples, respectively [81,92,93]. Although the LODs are all lower than 10 ng/mL, there are also inevitable disadvantages, including cumbersome pretreatment steps and complicated sample screening. To further improve the operation steps and broaden the application fields of the GICA method, Byzova et al. detected STR in milk and dairy products by a rapid and pretreatment-free immunochromatography. The target compound residue was measured based on the competition between immobilized STR-protein conjugates and STR in the real sample. Compared with the traditional ELISA, the quantitative analysis results of this method had a good correlation with the r values of 0.935 and 0.940, respectively [94]. To a certain extent, this detection method shortens the detection time, cuts cost and provides a technical support for the GICA detection of STR in foods. Although this detection method is convenient and fast, there is still a lot of development space for the synthesis of markers.

To further optimize the synthesis of markers, signal enhancement strategies are gaining more and more attention. Pang et al. used the one-pot method to synthesize CG@PDA and established an enhanced immunochromatography (ICA) based on colloidal gold-modified polydopamine (CG@PDA) for the simultaneous determination of GEN in milk, muscle, liver and kidneys. Compared with the traditional GICA, the sensitivity of the CG@PDA-ICA method was increased by 92 times [95]. Furthermore, it also possesses stronger signal intensity, better colloidal stability and a higher antibody coupling rate. Studies have shown that PDA is an effective signal-boosting substrate that can improve detection performance and offer a new technical platform for residue monitoring of GEN or other chemical contaminants.

For the sake of improving the detection efficiency, GICA is no longer limited to the detection of a single sample and multiple detection strategies have stepped onto the technical stage. Zhou et al. established a quadruple GICA for the simultaneous determination of multiple antibiotics, including β-lactam, tetracyclines, STR and chloramphenicol in milk. In the qualitative analysis, the visual cut-off value of STR was 50 ng/mL, the detection range was 0.78 ng/mL–25 ng/mL and the recovery rate was 84.5–107.6% [96]. In addition, the GICA method has been proved to be consistent with the instrumental analysis. This multiple detection strategy allows GICA to identify AGs residues in foods faster, more accurately and more efficiently, making it a promising method for rapid screening detection.

Colloidal gold labeling has little effect on the activity of biomolecules. It is an ideal marker for a lot of biomolecules in the immunoassay. As the advantages of GICA have been continuously explored, it has gradually been widely used in biology, immunology, antibiotic residues detection and other fields. At the same time, there are some problems existing, such as low stability, sensitivity and specificity of the colloidal solution, poor permeability owing to the large particles and high requirements sin the operating cleanliness degree. With the development of modern biotechnology and nanomaterials, quantum dots, magnetic nanoparticles, lanthanide elements, carbon nanoparticles, liposomes and other new labeling materials are gradually improving the performance of the colloidal gold immunoassay. The combination of colloidal gold technology and nanomaterials will become a new direction for the development of immunoassay technology.

### 4.3. Chemiluminescence Immunoassay

Chemiluminescence immunoassay (CLIA) is also known as chemiluminescence labeled immunoassay. This method is used to directly label the chemiluminescent substance on the antigen or antibody to detect the target compounds through a specific reaction. After oxidant or enzyme luminescent substrate is added to the mixture, an intermediate (excited state) is generated with energy releasing. Then, quantitative or qualitative detection can be performed with the luminescence intensity. Luminol, isoluminol and their derivatives are commonly used for labeling. According to the different markers, it is mainly divided into direct chemiluminescence immunoassay (DCLIA) and chemiluminescence enzyme immunoassay (CLEIA). Because of its high sensitivity, strong specificity, simple operation and no radioactive hazards, CLIA has been widely used in various fields involving antibiotic residue analysis, environmental monitoring and clinical diagnosis [97].

#### 4.3.1. Direct Chemiluminescence Immunoassay

DCLIA refers to the chemiluminescence of a labeled antibody/antigen reacting with the corresponding antigen/antibody to form an immobile coated antibody-antigen complex in the sample. Next, oxidant (H_2_O_2_) and NaOH are added to create an alkaline environment. Subsequently, the chemical luminant is decomposed and emits light. The photon energy generated per unit of time is received and recorded by the light collector and photomultiplier tube. Then, the content of antigen or antibody can be obtained by the calculation. In the DCLIA, luminol and acridine esters are commonly used luminol systems.

In recent years, multiple detection strategies have been paid more and more attention in the application of DCLIA [98,99]. Zeng et al. designed a nitrocellulose membrane-based microarray format to establish a simple and rapid multiple direct competitive chemiluminescence (CL) imaging immunoassay. They used this method to detect KAN and STR simultaneously. The results showed that the LODs of KAN and STR were 0.03 ng/mL and 0.33 ng/mL, respectively [100]. Furthermore, this method meets the requirements of MRLs. However, it is limited in that only two targets can be measured simultaneously. In addition, nanomaterials are being gradually applied with the multiple detection format in order to detect three or more targets simultaneously. Zeng et al. used the single-walled carbon nanotube (SWCNT) as the carrier, fixed with the coated antigen on the nitrocellulose membrane and then established a nitrocellulose-membrane based chemiluminescence microarray immunoassay. This method was used for the AGs residue analysis. The LODs were determined as 0.38 ng/mL, 0.64 ng/mL, 0.39 ng/mL and 1.25 ng/mL for GEN, KAN, NEO and STR/DHS in milk, respectively [101]. Compared with the traditional CLMIA, the LODs were increased by 11, 7, 7 and 3 times, respectively. Furthermore, this method had no cross-reactivity with other AGs and antibiotics and the inter-batch differences were in a range between 2.18 and 18.57%. It has high specificity, stability and reproducibility. It provides an effective and robust tool for the simultaneous detection of multiple residues in foods of animal origin.

In the DCLIA detection, the multiple detection strategy not only shortens the detection time and simplifies the operation steps, but also provides effective and reliable technical support for the accurately and precisely detection of multiple AGs targets in foods. However, enhancing the signal-noise ratio and reducing the signal interference are still the core tasks in the determination. By introducing new identification elements and marking materials, the signal strength of DCLIA can be enhanced and the detection performance of AGs residues can be improved. In addition, efficient and sensitive detection of multiple AGs in the same matrix is also a new development trend for DCLIA. We still need to continuously refine and improve the DCLIA method to make a certain contribution to the detection of AGs residues.

#### 4.3.2. Chemiluminescence Enzyme Immunoassay

CLEIA is based on the specific reaction of the enzyme-labeled antigen or antibody with the corresponding antibody or antigen to form a complex in the sample. After washing, the substrate (luminant) is added. Then, the enzyme catalyzes and decomposes the substrate and emits luminescence. The CLEIA determination can be performed by measuring the optical signal. At present, the commonly used labeling enzymes are rye root peroxidase (HRP) and alkaline phosphatase (ALP).

In many of the literature reports, the residue analysis of AGs in different food matrices is usually carried out by ELISA, aptamer sensing detection, immunochromatography and other methods. However, CLEIA is rarely used due to its unstable fluorescence signal. To improve the analytical ability of CLEIA for AGs residues, Luo et al. developed a sensitive CLEIA for the determination of trace amounts of NEO residues in milk. The IC_50_ value of CLEIA was 2.4 ng/mL, the LOD was 9.4 μg/kg and the recovery was 88.5–105.4% [102]. The results showed that the sensitivity has been improved by at least two to three orders of magnitude, which can be used for the rapid screening of NEO residues in milk. On the basis of CLEIA, the effects of direct- and indirect- detection models on this method are further explored. Li et al. established a sensitive and rapid chemiluminescence indirect enzyme immunoassay (CL-ciELISA) for the detection of GEN residues in milk. The IC_50_ of this method for GEN was 0.69 ng/mL and the LOD was 0.06 ng/mL [103]. Compared with the traditional colorimetric method, the sensitivity of CL-ciELISA based on a luminol solution was several times higher. This method was developed with the luminol-hydrogen peroxide-enhancer system. It is of great significance for controlling AGs contaminated milk samples.

CLEIA has the advantages of high sensitivity, good specificity, wide linear range and simple operation. It has also been widely used in clinical testing, drug analysis and other fields and has gradually become one of the analysis tools for food safety detection. In the future, researchers should make great efforts to develop multi-target detection, reduce interference of background signals and improve the luminant stability. By continuously improving the analytical performance of CLEIA, it will possess a broader application prospect in the field of animal-derived food safety inspection and it is of great significance to safeguarding people’s health.

### 4.4. Aptamer Sensing Detection

By using specific recognition elements, sensor detection has been widely used in the detection of antibiotic residues [104]. Aptamer sensing detection is a class of biosensors that are based on specific recognition between antigens and antibodies on the sensor surface [105]. It has attracted wide attention because of its simple structure, flexible application, convenient use and the amenability of integration into multi-functional analysis [106,107,108]. In addition, specific recognition provides the unique selectivity and high sensitivity for the sensor [109], which makes it gradually applied to the fields of food safety control [110,111,112,113,114], environmental monitoring [115,116,117] and medical diagnosis [118,119,120].

#### 4.4.1. Establishment of Detection Methods

**Figure 3:**
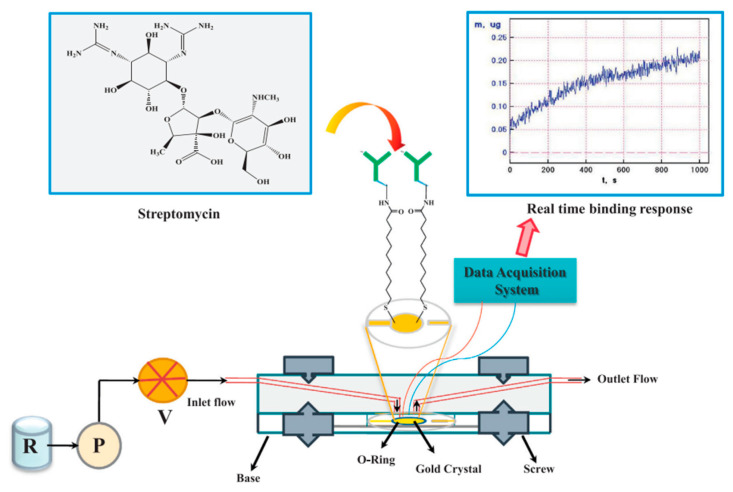
Schematic of flow injection analysis–electrochemical quartz crystal nonequilibrium (FIA-EQCN) biosensor for analysis of streptomycin in milk [122] (Reproduced with permission from Elsevier Ltd.).

Recognition components and signal amplification strategies play a critical role in the sensor detection. Ou et al. used a multi-cycle signal amplification strategy and incorporated with double-strand displacement and DNA three-way ligation structure to establish a highly sensitive and selective colorimetric sensor for TOB. The LOD of this method was 12.24 nmol/L and the linear range was 20 nmol/L–800 nmol/L [121]. The detection process avoids the unfavorable factors of expensive consumables and complex preparation. It contributed to the detection of TOB residues in milk and water samples. Mishra et al. integrated flow injection analysis-electrochemical quartz crystal nonequilibrium (FIA-EQCN) technology and immunological technology to prepare a sensor for the detection of STR residues (Figure 3). The correlation coefficients (R^2^) of this method were 0.997 and 0.994 in milk and PBS, respectively [122]. The developed FIA-EQCN sensor has good reproducibility with a relative standard deviation of 0.351% (n = 5). It can be used for the trace analysis of STR in milk samples. To further investigate the effect of the modified RNA aptamer on the affinity of NEO B, Noemi et al. constructed a novel SPR sensor for the detection of small molecules based on methylated RNA aptamer. According to the response values of the SPR sensor, the concentration of NEO B, the dissociation constant and the stoichiometry value of the aptamer can be measured indirectly. In addition, the linear detection range was 10 nmol/L–100 μmol/L and the LOD was 5 nmol/L [123]. This method provides a new reference and insight for the high-performance sensing detection of AGs residues.

#### 4.4.2. Application of Novel Signal Labels

Nanomaterials with outstanding catalytic properties are widely used in the construction of immunosensors to improve electrode conductivity and facilitate electron transfer [106,124]. They play an important role in the construction of immunosensor [109]. Xu et al. designed a new type of nucleic acid aptamer sensor based on an analyte-protection AgNPs probe and the selective recognition. This method could detect KAN residues in milk within 20 min. The linear concentration range was from 0.05 g/mL to 0.6 g/mL and the LOD was up to 2.6 ng/mL [125]. It can greatly shorten the detection time while ensuring sensitivity and can effectively eliminate the potential interference caused by the coexistence of protective agents in complex samples. The detection strategy in this model can further improve the detection performance and better meet the monitoring of trace antibiotic residues.

Based on the application of single nanomaterials, aptamer sensing strategies for multifunctional composite materials have also been gradually developed. Yin et al. used AuNPs and functional composites to conduct an in-depth study of GCE and constructed a simple and sensitive EC aptamer-selective sensor with porous carbon nanorods as the core conductive material. Under the optimal conditions, the LOD was 0.036 ng/mL and the linear range was 0.05 ng/mL–300 ng/mL [126]. This method can be used to detect STR residues selectively and specifically in milk and honey. Currently, nanomaterials such as gold nanoparticles [127], gold nanorods [128], microporous carbon spheres [129], nanostructured minerals [130], metal oxide nanofibers [131] and metal-organic frameworks are gradually used to construct the sensors. Using nanocomposites to modify sensors endows the detection with more sensitivity, accuracy, credible and greater convenience. The incorporation of modified nanomaterials and sensing technology not only makes the recognition performance superior, but also provides an excellent tool for the residue analysis.

Aptamer sensing assays, combining the specificity of biorecognition elements with the high sensitivity of sensors, have been widely used in the quantitative detection of AGs residual contamination. Although this technology has many advantages, it still has some limitations and challenges. First of all, further research is needed on the preparation of stable and specific antibodies for large-scale and industrial applications. Furthermore, multi-channel detection strategies should be investigated to achieve simultaneous detection of multiple targets on a single sensing interface. In addition, the application of nanomaterials in aptamer sensing needs to be further studied. It is believed that with the continuous improvement of scientific research technology and the unremitting efforts of researchers, aptamer sensing detection will become a rapid and effective analytical tool for AGs residues detection. Table 5 summarizes the application of KAN aptamer sensing detection in the animal-derived foods. By using the aptamer analysis method, it can quickly and accurately detect AGs residues in honey, milk and meat samples. With the introduction of new signal tags, the performance of aptamer analysis has been greatly improved. In addition, this technology provides a new idea for improving the sensitivity, stability and practicability of the AGs residue detection.

### 4.5. Fluorescence Immunoassay

Fluorescence immunoassay (FIA) is a method by which the specificity of immunological responses is combined with the sensitivity of fluorescence techniques. Then, by the specific fluorescence imaging, the quantitative analysis can be performed and achieved.

Quantum dots [136], upconversion phosphors [137] and superparamagnetic beads [138] nanomaterials have been widely used in fluorescence immunoassay to achieve quantitative detection of AGs. Song et al. combined fluorescence immunoassay technology based on multicolor quantum dots (QD) with an array analysis method to detect the STR residues in milk. STR was conjugated with QD as a detection probe and then followed by a direct competitive fluorescence immunoassay in an antigen-coated microtiter plate. The linear range of STR was determined from 0.01 ng/mL to 25 ng/mL and the LOD was 5 pg/mL [139]. This method achieved a pico-level analysis of STR residues in foods. Although nanomaterials provide simple and rapid residue detecting procedures, some complex sample matrices may interfere with specific recognition and lead to ambiguous results.

Europium (Eu) (III), terbium (Tb) (III), samarium (Sm) (III), dysprosium (Dy) (III) and other lanthanides can effectively eliminate the non-specific interferences of the fluorescence background signals and improve the sensitivity owing to their long fluorescence lifetime and large stokes shift [140,141]. Wang et al. prepared a STR monoclonal antibody in conjugation with Eu (III) nanoparticles as the probe and established the chromatographic time-resolved fluoroimmunoassay (CTRFIA) for STR residue measurement in milk. The LOD of this method was 0.58 µg/kg, the linear range was from 0.8 µg/kg to 6.25 µg/kg and the recovery was from 85.6% to 108.3% [142]. As shown by the data, the lanthanides can greatly reduce the interference of background signals and improve sensitivity. In addition, lanthanides make up for the shortage of nanomaterials to a certain extent. They are more conducive to controlling the hazards of antibiotic residues.

In order to improve the analysis efficiency, a method for simultaneous detection of multiple analytes has gradually become an urgent need for the screening of AGs residues in foods. With the development of the fluorescent immunoassay, the suspension array technology can simultaneously recognize and quantify up to 100 different targets at the single molecule level. Wang et al. developed a bead-based indirect competitive fluorescent immunoassay using Bio-Plex ^TM^ 200 suspension array technology for the rapid detection of AGs residues in the animal-derived foods. The results showed that the LODs of KAN and GEN were 0.5 ng/mL and 4.1 ng/mL in PBS, and 2.2 ng/mL and 12.2 ng/mL in milk, respectively. There was no significant cross-reaction with other AGs [143]. As a time-saving, simple and effective detection method, this method not only promotes the detection of AGs residues in practical applications, but also lays a solid foundation for the detection of multi-target analytes in foods.

Fluorescence immunoassay provides a new method and strategy for food safety detection due to its high throughput, simple operation and high sensitivity. However, there are still some shortcomings in the current fluorescence immunoassay. For example, the fluorescence materials are easily involved in a photobleaching phenomenon. Furthermore, quantum dots contain toxic heavy metal ions that are harmful to organisms and the environment. In addition, this method is easily disturbed by the sample matrix, resulting in great variations in parameters such as precision and accuracy. Moreover, labeling antibodies with the fluorescent substances may lead to non-specific staining. Additionally, the stability and specificity of fluorescent markers also have some influences on the detection results. Therefore, in order to solve the above problems, one possible way is to innovate and develop new recognition models and fluorescence materials to achieve highly sensitive and selective detection.

### 4.6. Biomimetic Sensing Detection Based on Molecularly Imprinted Polymers

Molecular imprinting technology (MIT), is now recognized as one of the most rapid and powerful methods for creating tailor-made synthetic polymers with strong and selective affinities for a diverse selection of analytes. Molecularly imprinted polymers (MIPs) can be prepared with the specific recognition sites for target molecules by the MIT [144]. Due to its advantages of low cost, easy preparation, reusability and high chemical stability, great progress has been made in the fields of biological recognition, membrane separation, chemical catalysis reaction and so on [145,146].

#### 4.6.1. Biomimetic Enzyme-Linked Immunoassay

The MIPs prepared by the MIT have similar recognition processes to biological antibodies. It is proved that the MIPs are ideal antibody substitutes for the development of affinity-based analysis. Tang et al. prepared MIPs by solid-phase synthesis, then determined GEN in milk by the biomimetic enzyme-linked immunoassay. In the detection, HRP-labled GEN and free GEN are competitively bound with the molecularly imprinted interface. The recovery test was carried out by spiking different concentrations of gentamicin (6 μmol/L, 18 μmol/L and 60 μmol/L) to milk with an average recovery rate of 94%. Meanwhile, GEN nanoMIPs are specific for their target with little or no cross-reactivity with STR and ampicillin [147]. In order to further expand the application range of this technology, Zhang et al. detected KAN with the recognition element of MIPs in milk powder and honey. In addition, the LODs of the method were 4.33 × 10^−8^ mol/L and 1.20 × 10^−8^ mol/L, the relative standard deviation were 1.18% and 0.31%, respectively [51]. It showed that the method has good sensitivity and selectivity for the determination of KAN. Moreover, it is necessary to further study how to improve the specificity and sensitivity of the recognition elements, so as to provide better technical support for the AGs residues monitoring. Que et al. developed a molecular imprinting sensor for the detection of STR in honey and milk samples. This method is based on the competitive binding of glucose oxidase (GOx)-labeled STR and free STR at binding sites on the surface of a MIPs-modified transducer. The LOD was calculated as 7.0 mg/mL and the linear concentration range was from 0.01 mg/mL to 10 mg/mL [148]. The proposed new detection method has great theoretical significance and application prospects in food safety inspection.

#### 4.6.2. Biomimetic Electrochemistry Based on Magnetic Molecularly Imprinted Particles

**Figure 4:**
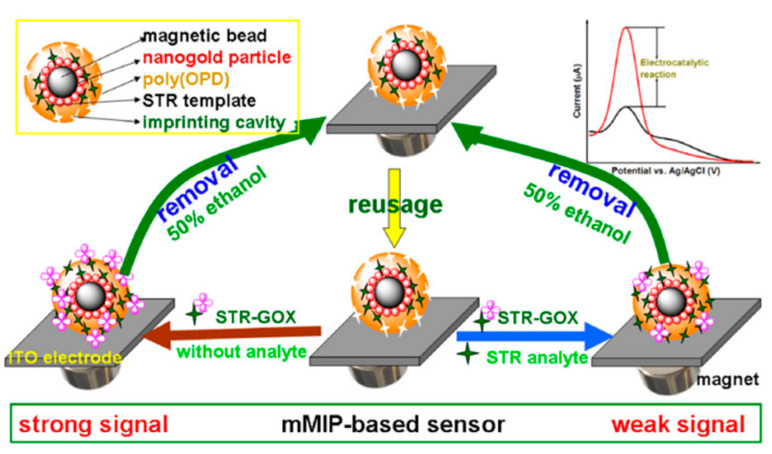
Schematic illustration of nanogold-promoted magnetic molecularly imprinted polymer nanospheres for competitive-type electrochemical detection of streptomycin (STR) residues by coupling with bioelectrocatalytic reaction of glucose oxidase (GOX) for signal amplification [149] (Reproduced with permission from Elsevier Ltd.).

The development of magnetic molecularly imprinted particles (MMIPs) is a new approach to improve the residue analysis with high selectivity and sensitivity and robust detecting performance. Liu et al. synthesized MMIPs with a redox active by one-pot method (Figure 4). This method is coupled with the bioelectrocatalytic reaction of enzymes for signal amplification to realize high-efficiency electrochemical detection of STR residues in foods. Under the optimal conditions, the linear range was from 0.05 ng/mL to 20 ng/mL, the LOD was calculated as 10 pg/mL and the intra- and inter-batch coefficients of variation were lower than 12% [149]. As an effective recognition element, MMIPs can rapidly and sensitively detect AGs residues in foods. In order to further improve the detection efficiency and broaden the application range of the technology, Zhang et al. prepared MMIPs for electrochemical sensing detection of TOB, GEN and KAN. This approach used metal ions as signal tracers and amplifiers for MMIPs. The quantitation limits of KAN, TOB and GEN were 16.27 nmol/L, 4.27 nmol/L and 3.57 nmol/L, respectively. In addition, the recoveries of KAN, TOB and GEN in milk were 96.8% to 104.5%, 98.5% to 104.8% and 95.4% to 103.5%, respectively [150]. The results revealed that this method had a relatively stable precision and good reproducibility. In this method, although the prepared MMIPs have high specificity and a strong capacity for recognition, the disadvantages of complex material modification and difficulty in large-scale preparation still hinder its development and popularization to a certain extent.

#### 4.6.3. Biomimetic Surface Plasmon Resonance

Integrating MIT technology with surface plasmon resonance (SPR) sensing detection is also an effective method for the analysis of trace compounds in complex matrices. Frasconi et al. developed a SPR based on the immunoassay method. They prepared biomimetic antibody membranes of NEO, KAN and STR by electropolymerization. This method was incorporated with the LSPR effect of AuNPs to further enhance the signal intensity. Furthermore, the LODs of NEO, KAN and STR were 2.00 pmol/L, 1.00 pmol/L and 200 fmol/L, respectively [151]. The incorporation of imprinted film and SPR technology makes the sensing detection with high sensitivity and good long-term stability. According to the related research, Han et al. detected KAN using carboxylated single-walled carbon nanotubes modified GCE with cyclic voltammetry. The sensor exhibited a linear range from 0.1 μmol/L to 50 μmol/L and a LOD of 0.1 μmol/L. Furthermore, this method had good reproducibility with a relative standard deviation of 3.12% (n = 6) [152]. Although this method has a high recognition ability, the sensitivity is not ideal in the field of antibiotic detection and needs to be further improved. In addition, the preparation process is complex and difficult to control. In order to improve the sensitivity and expand the detection range, Yu et al. introduced reduced graphene oxide (rGO) and poly (2-aminopyridine) (pAP) as the amplification materials to develop KAN electrochemical sensing detection. The linear range of this method for detecting KAN was from 0.02 μmol/L to 250.0 μmol/L and the LOD was 7.0 nmol/L. In addition, the recoveries were from 89.2% to 109.0% with a relative standard deviation of less than 6.0% for the determination of KAN in pork and milk by this method [153]. This method has good analytical performance in terms of selectivity, stability, sensitivity and repeatability. As an effective recognition element, MIPs-based analysis methods can be used to detect small molecule contaminants in foods. This method provides a new technical tool for food safety monitoring and detection.

In recent years, molecular imprinting detection has been developed rapidly, but there are still some challenges for its application in the field of food safety inspection. For example, incomplete elution or leakage of template molecules of the MIPs can result in trace amounts of the target compounds being present in the analysis. In addition, food testing is usually performed in aqueous solutions, but MIPs suitable for aqueous solutions are limited. Moreover, this technique is less studied for complex samples. These deficiencies all restrict the development of molecular imprinted biomimetic assay. Therefore, increased scientific research efforts should be funded to improve the analysis performance to make great contributions to food safety monitoring.

### 4.7. Lateral Flow Immunoassay

Lateral flow immunoassay (LFA), also known as immunochromatography (ICA), is based on the specific binding reactions of antigens and antibodies with high affinity and specificity. Compared with the traditional immunoassay, this technology has the advantages of simplicity, rapidity and low cost. In addition, the detection time of ICA is usually within 10–20 min. It can also be used for high-throughput detection of large-scale samples, especially used in the field of food safety [154,155].

#### 4.7.1. Visible Lateral Flow Immunoassay

Applying LFA technology to sensors can have a certain impact on the detection results. Liu et al. developed a lateral flow strip shaped immunosensor to detect KAN. In this method, the detection was performed by using aptamer-modified gold nanoparticles as the probe and oligonucleotide DNA 1-modified silver nanoparticles as the signal amplification element. This method achieved visual qualitative detection of KAN within 10 min. The linear detection range was from 1 nmol/L to 30 nmol/L and the minimum LOD was 0.0778 nmol/L [156]. Moreover, combining biological barcode technology with affinity-sensing detection, this method has good sensitivity, specificity and stability. However, it is found that the limited factors, including the cumbersome material preparation process and expensive special consumables, hinder the popularization and application of this method to a certain extent.

#### 4.7.2. Lateral Flow Immunoassay Based on Nano-Enzymes

**Figure 5:**
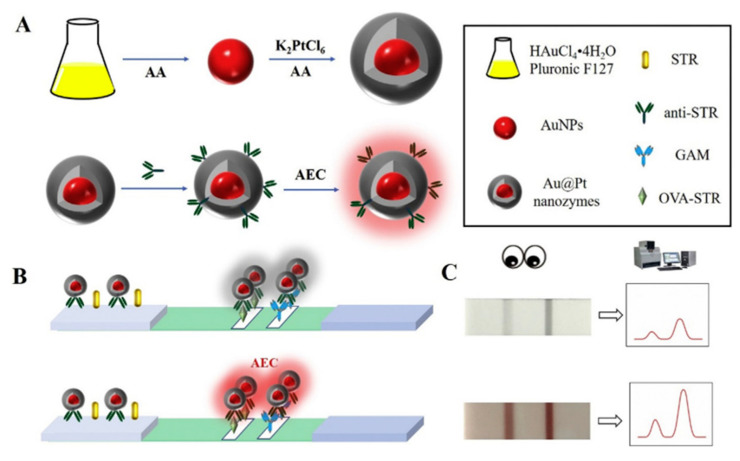
Schematic illustration for the colorimetric lateral flow immunoassay (LFA) based on Au@Pt. (**A**) Preparation of Au@Pt nanozyme; (**B**) Development of the colorimetric LFA based on Au@Pt; (**C**) Typical results of the colorimetric LFA assay based on Au@Pt [157] (Reproduced with permission from Elsevier Ltd.).

LFA can effectively improve the sensitivity by using time-resolved fluorescent microspheres (TRFM) [142], quantum dots [136], Au@Pt [157] and other signal labels. The application of nanomaterials also has a certain influence on the test results. Hendrickson et al. compared the effect of direct and indirect applications of AuNPs as the labels on the LFIA detection results of NEO. The LOD of NEO by indirect method was 0.1 ng/mL, the linear range was 0.28 ng/mL–5.18 ng/mL and the cutoff value was 10 ng/mL [75]. The results showed that the indirect method increased the sensitivity by 80 times compared with the direct method. This technique allows for the NEO recoveries of 70–119% in milk, honey, turkey meat and eggs (coefficient of variation 10%). Additionally, to improve efficiency and reduce cost, bimetallic nanozyme strategies have been developed and used for AGs residue detection. Wei et al. developed a lateral-flow immunoassay based on bimetallic nano-enzymes Au@Pt and AuNPs for the detection of STR residues in cow milk (Figure 5). Compared with the traditional LFA based on single metal AuNPs or Au@Pt, the LFA based on bimetallic AuNPs and Au@Pt increases the LOD by 8 and 80 times, respectively [157]. This study shows that the bimetallic nanozyme-based LFA can give full play to its unique advantages, improve the LOD and sensitivity and expand the application range.

#### 4.7.3. Lateral Flow Immunoassay Based on Fluorescent Materials

Although signal labeling can improve the sensitivity of LFA, unfortunately, the detection method is still limited to the detection of AGs residues in partial foods of animal origin. In order to expand the application scope of this method, Jiang et al. established a time-resolved fluorescence microsphere lateral-flow immunoassay (TRFM-LFIA) with a portable fluorometer for rapid detection of STR and DHS in the foods of animal origin [158]. With this method, the sample pretreatment is very simple and can be completed quickly in 8 min. This rapid, sensitive, simple and portable analysis method can provide powerful technical support for the residual detection of antibiotics or even more small molecules. On the basis of the detection model, Liu et al. developed a sensitive and reliable flow immunoaffinity chromatography assay (FTIACT) for rapid analysis of NEO B in milk [159]. The established FTIACT method can detect NEO B at the femoral level in foods, shorten the detection time and be performed in any routine laboratory.

Due to its portability, preparation and timeliness, LFA has been widely used in the detection of AGs residues and developed new prevention and control measures for monitoring food safety issues. However, there are still a number of areas that need to be improved, including many sample extraction steps and low repeatability of complex matrix detection. Simplified sample pretreatment can reduce matrix background interference and help improve the sensitivity and reproducibility of LFA. Furthermore, most lateral flow immunoassays only provide qualitative or semi-quantitative results. It can not meet the requirements of food safety inspection. By using new recognition elements and labeling materials, the analytical performance was highly improved in the LFA detection. Moreover, some signaling labels such as quantum dots, heavy metals and fluorescent labels may be harmful to the environment when used on a large scale. Therefore, we still need to constantly overcome the difficulties and challenges encountered in the detection process.

## 5. Conclusions and Prospects

Affinity-based analysis methods stand out among many detection methods due to their unique advantages such as high sensitivity, strong specificity and simple operation. Representative analytical methods, including ELISA, LFA, CLIA and GICA, have been widely used in food safety monitoring. By using antibodies, aptamers and MIPs as the recognition elements, and precious metal materials and nano-enzyme as the sensitizing materials, direct or indirect detection models and simple sample pretreatment methods including SPE, PLE and MSPD can further improve AGs analytical performance. Combining these above methods with nanotechnology and spectroscopic technology is becoming a new development trend, making for faster, more sensitive and more efficient detection of AGs. They provide efficient and reliable technical tools to promote food safety and human health.

Exploring affinity analysis methods with multiple detection capabilities is a developing trend to meet higher detection requirements. Therefore, some related suggestions are put forward. First, the development of recognition elements with high specificity, high sensitivity and high affinity is essential for analysis [160] since the food matrix is a complex system containing various interferences [161]. It will affect the detection performance of antibiotic residues. Recognition elements with broad specificity and high affinity for multiple targets should be designed and obtained [162]. Moreover, the design of new portable instruments and the synthesis of new functional materials can facilitate AGs detection by the affinity-sensing analysis. Second, the preparation of stable and specific novel signaling tags is necessary to improve the accuracy and sensitivity of the detection. Developing nanomaterials with better performance to improve the response to subtle concentration changes will be an important research direction in the future [163]. In addition, with the increase in the types and quantities of hazards to which foods are exposed, multiple detection technologies are also an inevitable tendency in the development of affinity analysis. Mixing different recognition probes to identify different targets on a single sensing platform seems to facilitate multi-target detection [164]. Future research and development should focus on integrating affinity-based sensing analysis technology with other new technologies to achieve a highly sensitive, rapid, miniaturized and integrated detection of AGs residues. Therefore, it is a highly important requirement to continuously strengthen scientific research innovation and development of affinity-based analysis methods. This will make great contributions to food safety inspection and human health.

## Figures and Tables

**Table 1:** Maximum residue limits (MRLs) in different countries.

Aminoglycoside	Animal	Target	MRL (µg/kg)			
			America	European Union	China	Japan
NEO	Pork	Muscle	1200	500	500	500
		Fat	-	500	500	500
		Liver	3600	500	5500	500
		Kidney	7200	5000	9000	10,000
	Cattle/sheep	Muscle	1200	500	500	500
		Fat	7200	500	500	500
		Liver	3600	500	5500	500
		Kidney	7200	5000	9000	10,000
		Milk	150	1500	1500	2000
	Chicken	Muscle	-	500	500	500
		Liver	-	500	5500	500
		Kidney	-	5000	9000	10,000
GEN	Pork	Muscle	100	50	100	100
		Fat	400	50	100	100
		Liver	300	200	2000	2000
		Kidney	400	750	5000	5000
	Cattle	Muscle	-	50	100	100
		Liver	-	200	2000	2000
		Kidney	-	750	5000	5000
		Milk	-	100	200	200
	Chicken	Muscle/fat/kidney	100	-	100	-
KAN	Pork	Muscle/fat	-	100	100	40
		Liver	-	600	600	900
		Kidney	-	2500	2500	4000
	Cattle	Muscle/fat	-	100	100	40
		Liver	-	600	600	1000
		Kidney	-	2500	2500	13,000
		Milk	-	150	150	700
	Sheep	Muscle/fat	-	100	100	100
		Liver	-	600	600	600
		Kidney	-	2500	2500	3000
	Chicken	Muscle	-	100	100	200
		Fat	-	100	100	300
		Liver	-	600	600	1300
		Kidney	-	2500	2500	25,000
STR	Pork	Muscle/fat/liver	500	500	600	600
		Kidney	2000	1000	1000	1000
	Cattle	Muscle/fat/liver	500	500	600	600
		Kidney	2000	1000	1000	1000
		Milk	500	200	200	200
	Sheep	Muscle/fat/liver	-	500	600	600
		Kidney	-	1000	1000	1000
	Chicken	Muscle/fat/liver	500	-	600	600
		Kidney	2000	-	1000	1000
SPE	Pork	Muscle	-	300	500	500
		Fat	-	500	2000	2000
		Liver	-	1000	2000	2000
		Kidney	-	5000	5000	5000
	Cattle	Muscle	2500	300	500	500
		Fat	-	500	2000	2000
		Liver	-	1000	2000	2000
		Kidney	4000	5000	5000	5000
		Milk	-	200	200	200
	Sheep	Muscle	-	300	500	500
		Fat	-	500	2000	2000
		Liver	-	2000	2000	2000
		Kidney	-	5000	5000	5000
	Chicken	Muscle	100	300	500	500
		Fat	100	500	2000	2000
		Liver	100	1000	2000	2000
		Kidney	100	5000	5000	5000
DHS	Pork	Muscle/fat/liver	500	500	600	600
		Kidney	2000	1000	1000	1000
	Cattle	Muscle/fat/liver	500	500	600	600
		Kidney	2000	1000	1000	1000
		Milk	125	200	200	200
	Sheep	Muscle/fat/liver	-	500	600	600
		Kidney	-	1000	1000	1000
	Chicken	Muscle/fat/liver	-	-	600	600
		Kidney	-	-	1000	1000

-: not found.

**Table 2:** Sample pretreatment methods for AGs residue analysis.

Pretreatment	Sample Matrix	Detection Method	AGs	Reference
Dissolve and dilute	Commercial samples	CE-C^4^D	TOB	[46]
SPME	Milk and honey	LC-MS/MS	AMK, TOB DHS, KAN	[47]
	Tilapia	LC-ELSD	TOB, STR, NEO	[48]
MSPD	Milk powder and milk samples	LC-MS/MS	AMK, STR, DHS, KAN, GEN	[45]
SPE	Bovine edible tissues	LC-MS/MS	AMK, STR, DHS, KAN, NEO, GEN	[49]
	Milk	LC-MS/MS	STR, DHS	[50]
	Honey, milk powder	Spectrophotometry	KAN	[51]
	Milk and meat	LC-MS/MS	GEN, NEO, KAN, STR, DHS, AMK	[52]
PLE	Meat	LC-MS/MS	DHS, STR	[44]
DLLME	Milk	LC-FLD	NEO, TOB, GEN	[53]
Deproteinization	Milk and chicken egg	Colorimetric aptasensor	TOB	[54]
LLE	Milk and muscle	LC-MS/MS	TOB, GEN, KAN, STR, DHS, NEO	[17]
	Milk	FPIA	GEN	[43]
Extraction and dilution	Egg and milk	Nanosensor	TOB	[54]

**Table 3:** Comparison of advantages and disadvantages of affinity-based analysis methods.

Method	Advantage	Disadvantage
Enzyme-linked immunosorbent assay	Fast, sensitive, simple, high-throughput detection	Poor repeatability, high false negative results
Colloidal gold immunochromatographic assay	Wide application range, stable marker, low cost	Low stability, sensitivity and specificity of the colloidal solution
Chemiluminescence immunoassay	High sensitivity, strong specificity, simple operation, no radioactive hazards	Interference of background signals
Aptamer sensing detection	Simple structure, flexible application, convenient use	Low stability of the identification elements
Fluorescence immunoassay	Simple operation, high sensitivity	Photobleaching phenomenon of the fluorescence materials; heavy metal ions are harmful to the environment
Biomimetic sensing detection based on molecularly imprinted polymers	Low cost, easy preparation, reusability and high chemical stability	Incomplete elution or leakage of template molecules; MIPs of aqueous phase are limited
Lateral flow immunoassay	High affinity and specificity, simplicity, rapidity and low cost	Low repeatability, effect of sample matrix

**Table 4:** Application of ELISA detection of AGs residues in the animal-derived foods.

AGs	Matrix	Sensitivity	LOD	Recovery	Reference
Kanamycin	Milk, egg	0.65 ng/mL	0.28 ng/mL	73.55–84.61% 73.70–105.75%	[87]
Gentamicin, Kanamycin	Egg	0.32 μg/mL	0.001 μg/mL	55.34–133.51%	[73]
Gentamicin	Milk, pork, chicken	2.69 ng/mL	0.01 ng/mL	69.82–94.32%	[88]
Apramycin	Meat, egg	0.41 ng/mL	0.15 ng/mL	79.02–105.49%	[89]
Gentamicin	Meat, egg, milk	0.3 ng/mL	0.03 ng/mL	69–118%	[90]
Amikacin	Milk	1.30 ng/mL	11.3 ng/mL	69.8–93.3%	[91]

**Table 5:** Detection of kanamycin in animal food by affinity-based analysis method.

Detection Method	Matrix	LOD	Recovery	Linear Range	Time	Reference
GS-Nf/TH/Pt modified GCE	Chicken	5.74 pg/mL	99.4–106%	0.01 ng/mL–12.0 ng/mL	-	[132]
Colorimetric detection based on AuNPs	Meat, milk	2.6 ng/mL	-	0.05 µg/mL–0.6 µg/mL	20 min	[125]
Immunosensing detection based on biotin-streptavidin system	Milk	0.1 ng/mL	80.2–85.6%	1.5 ng/mL–25.2 ng/mL	45 min	[133]
Biomimetic SPR	Milk powder, honey	4.33 × 10^−8^ mol/L 1.20 × 10^−8^ mol/L,	91.6%, 99.8%	1.00 × 10^−7^ mol/L–1.00 × 10^−5^ mol/L	-	[51]
Electrochemical sensing detection	Serum, milk, water	3.3 pmol/L	77.3–97.3%	10 pmol/L–1.0 μmol/L	9 min	[134]
EIS based KANA-aptasensor detection	Milk	0.11 ng/mL	96.88–100.5%	1.2 ng/mL–75 ng/mL	60 min	[135]

-: not found.

## Data Availability

Data is contained within the article.

## References

[1] Yang B., Wang L., Luo C., Wang X., Sun C. (2017). Simultaneous determination of 11 aminoglycoside residues in honey, milk, and pork by liquid chromatography with tandem mass spectrometry and molecularly imprinted polymer solid phase extraction. J. AOAC Int..

[2] Stead D.A. (2000). Current methodologies for the analysis of aminoglycosides. J. Chromatogr. B.

[3] Turnipseed S.B., Clark S.B., Karbiwnyk C.M., Andersen W.C., Miller K.E., Madson M.R. (2009). Analysis of aminoglycoside residues in bovine milk by liquid chromatography electrospray ion trap mass spectrometry after derivatization with phenyl isocyanate. J. Chromatogr. B.

[4] Schilling K., Krmar J., Maljurić N., Pawellek R., Protić A., Holzgrabe U. (2019). Quantitative structure-property relationship modeling of polar analytes lacking UV chromophores to charged aerosol detector response. Anal. Bioanal. Chem..

[5] Ji S., Zhang F., Luo X., Yang B., Jin G., Yan J., Liang X. (2013). Synthesis of molecularly imprinted polymer sorbents and application for the determination of aminoglycosides antibiotics in honey. J. Chromatogr. A.

[6] Toro J., Rodríguez C.A., Zuluaga A.F. (2019). Effectiveness of the antibiotic combinations for enterococcal infections treatment: A critical review. Rev. Chil. Infectol..

[7] Luan Y., Wang N., Li C., Guo X., Lu A. (2020). Advances in the application of aptamer biosensors to the detection of aminoglycoside antibiotics. Antibiotics.

[8] Wang X., Yang S., Li Y., Zhang J., Jin Y., Zhao W., Zhang Y., Huang J., Wang P., Wu C. (2018). Optimization and application of parallel solid-phase extraction coupled with ultra-high performance liquid chromatography-tandem mass spectrometry for the determination of 11 aminoglycoside residues in honey and royal jelly. J. Chromatogr. A.

[9] Prayle A., Watson A., Fortnum H., Smyth A. (2010). Side effects of aminoglycosides on the kidney, ear and balance in cystic fibrosis. Thorax.

[10] Wu S., Zhang H., Shi Z., Duan N., Fang C., Dai S., Wang Z. (2015). Aptamer-based fluorescence biosensor for chloramphenicol determination using upconversion nanoparticles. Food Control.

[11] Saluti G., Diamanti I., Giusepponi D., Pucciarini L., Rossi R., Moretti S., Sardella R., Galarini R. (2018). Simultaneous determination of aminoglycosides and colistins in food. Food Chem..

[12] Berruga M., Molina A., Althaus R.L., Molina M. (2016). Control and prevention of antibiotic residues and contaminants in sheep and goat’s milk. Small Rumin. Res..

[13] El Hawari K., Mokh S., Doumyati S., Al Iskandarani M., Verdon E. (2017). Development and validation of a multiclass method for the determination of antibiotic residues in honey using liquid chromatography-tandem mass spectrometry. Food Addit. Contam. Part A Chem. Anal. Control Expo. Risk Assess..

[14] Perkons I., Pugajeva I., Bartkevics V. (2018). Simultaneous screening and quantification of aminoglycoside antibiotics in honey using mixed-mode liquid chromatography with quadrupole time-of-flight mass spectroscopy with heated electrospray ionization. J. Sep. Sci..

[15] Gajda A., Nowacka-Kozak E., Gbylik-Sikorska M., Posyniak A. (2019). Multi-residues UHPLC-MS/MS analysis of 53 antibacterial compounds in poultry feathers as an analytical tool in food safety assurance. J. Chromatogr. B.

[16] Kumar P., Rubies A., Companyó R., Centrich F. (2012). Determination of aminoglycoside residues in kidney and honey samples by hydrophilic interaction chromatography-tandem mass spectrometry. J. Sep. Sci..

[17] Arsand J.B., Jank L., Martins M.T., Hoff R.B., Barreto F., Pizzolato T.M., Sirtori C. (2016). Determination of aminoglycoside residues in milk and muscle based on a simple and fast extraction procedure followed by liquid chromatography coupled to tandem mass spectrometry and time of flight mass spectrometry. Talanta.

[18] Dasenaki M.E., Michali C.S., Thomaidis N.S. (2016). Analysis of 76 veterinary pharmaceuticals from 13 classes including aminoglycosides in bovine muscle by hydrophilic interaction liquid chromatography-tandem mass spectrometry. J. Chromatogr. A.

[19] Ricker N., Trachsel J., Colgan P., Jones J., Choi J., Lee J., Coetzee J.F., Howe A., Brockmeier S.L., Loving C.L. (2020). Toward antibiotic stewardship: Route of antibiotic administration impacts the microbiota and resistance gene diversity in swine feces. Front. Vet. Sci..

[20] Reis A.C., Kolvenbach B.A., Nunes O.C., Corvini P.F. (2020). Biodegradation of antibiotics: The new resistance determinants—Part I. New Biotechnol..

[21] Sun X., Li F., Shen G., Huang J., Wang X. (2014). Aptasensor based on the synergistic contributions of chitosan-gold nanoparticles, graphene-gold nanoparticles and multi-walled carbon nanotubes-cobalt phthalocyanine nanocomposites for kanamycin detection. Analyst.

[22] Qin X., Guo W., Yu H., Zhao J., Pei M. (2015). A novel electrochemical aptasensor based on MWCNTs-BMIMPF 6 and amino functionalized graphene nanocomposite films for determination of kanamycin. Anal. Methods.

[23] Rosenberg C.R., Fang X., Allison K.R. (2020). Potentiating aminoglycoside antibiotics to reduce their toxic side effects. PLoS ONE.

[24] Mahi-Birjand M., Yaghoubi S., Abdollahpour-Alitappeh M., Keshtkaran Z., Bagheri N., Pirouzi A., Khatami M., Sineh Sepehr K., Peymani P., Karimzadeh I. (2020). Protective effects of pharmacological agents against aminoglycoside-induced nephrotoxicity: A systematic review. Expert. Opin. Drug Saf..

[25] Mingeot-Leclercq M.-P., Glupczynski Y., Tulkens P.M. (1999). Aminoglycosides: Activity and resistance. Antimicrob. Agents Chemother..

[26] Li Y.M., Zhang Y., Zhou Y., Liu Z.F., Meng Q., Feng X.S. (2021). Aminoglycosides in Food: Recent Updates on the Pretreatment and Analysis Methods. Food Rev. Int..

[27] Childs-Kean L.M., Shaeer K.M., Varghese Gupta S., Cho J.C. (2019). Aminoglycoside allergic reactions. Pharmacy.

[28] Fiekers J.F. (1983). Effects of the aminoglycoside antibiotics, streptomycin and neomycin, on neuromuscular transmission. I. Presynaptic considerations. J. Pharmacol. Exp. Ther..

[29] Selimoglu E. (2007). Aminoglycoside-induced ototoxicity. Curr. Pharm. Des..

[30] Zhou Y., Ji Y., Cao Z. (2020). Recent advances in optical detection of aminoglycosides. Appl. Sci..

[31] Jaimee G., Halami P. (2016). Emerging resistance to aminoglycosides in lactic acid bacteria of food origin-an impending menace. Appl. Microbiol. Biotechnol..

[32] Xu H., Tang C., Fan W. (2019). Research progress in aminoglycoside antibiotics. Chin. J. New Drugs.

[33] Foster II J., Tekin M. (2016). Aminoglycoside induced ototoxicity associated with mitochondrial DNA mutations. EJMHG.

[34] Xu L.J., Liu X., Zhang X.Q., Liu J.X., Miao C. (2016). Determination of aminoglycoside residues in eggs by UPLC-MS/MS. J. Pharm. Anal..

[35] Kang H.S., Kwon N.J., Jeong J., Lee K., Lee H. (2019). Web-based Korean maximum residue limit evaluation tools: An applied example of maximum residue limit evaluation for trichlorfon in fishery products. Environ. Sci. Pollut. Res..

[36] The European Commission (2010). Commission Regulation No. 37/2010 of 22 December 2009 on Pharmacologically Active Substances and Their Classification Regarding Maximum Residue Limits in Foodstuffs of Animal Origin. https://eur-lex.europa.eu/legal-content/EN/TXT/?uri=CELEX:32010R0037.

[37] The Japan Food Chemical Research Foundation (2019). Maximum Residue Limits List of Agricultural Chemicals in Foods. http://db.ffcr.or.jp/front/.

[38] US Food and Drug Administration (2019). US Food and Drug Administration CFR-Code of Federal Regulations Title 21 Part 556 Tolerances for Residues of New Animal Drugs in Food. https://www.accessdata.fda.gov/scripts/cdrh/cfdocs/cfCFR/CFRSearch.cfm?CFRPart=556%26showFR=1.

[39] Liu H., Li N., Liu X., Qian Y., Qiu J., Wang X. (2020). Poly (N-acryloyl-glucosamine-co-methylenebisacrylamide)-based hydrophilic magnetic nanoparticles for the extraction of aminoglycosides in meat samples. J. Chromatogr. A.

[40] Lou X., Tang Y., Fang C., Kong C., Yu H., Shi Y., Huang D., Guo Y., Xiao D. (2020). Simultaneous determination of ten aminoglycoside antibiotics in aquatic feeds by high-performance liquid chromatography quadrupole-orbitrap mass spectrometry with pass-through cleanup. Chirality.

[41] Savoy M.C., Woo P.M., Ulrich P., Tarres A., Mottier P., Desmarchelier A. (2018). Determination of 14 aminoglycosides by LC-MS/MS using molecularly imprinted polymer solid phase extraction for clean-up. Food Addit. Contam. Part A Chem. Anal. Control Expo. Risk Assess..

[42] Tarannum N., Khatoon S., Dzantiev B.B. (2020). Perspective and application of molecular imprinting approach for antibiotic detection in food and environmental samples: A critical review. Food Control.

[43] Beloglazova N., Shmelin P., Eremin S. (2016). Sensitive immunochemical approaches for quantitative (FPIA) and qualitative (lateral flow tests) determination of gentamicin in milk. Talanta.

[44] Berrada H., Moltó J.C., Mañes J., Font G. (2010). Determination of aminoglycoside and macrolide antibiotics in meat by pressurized liquid extraction and LC-ESI-MS. J. Sep. Sci..

[45] Wang L., Yang B., Zhang X., Zheng H. (2017). Novel two-dimensional liquid chromatography-tandem mass spectrometry for the analysis of twenty antibiotics residues in dairy products. Food Anal. Methods.

[46] El-Attug M.N., Hoogmartens J., Adams E., Van Schepdael A. (2012). Optimization of capillary electrophoresis method with contactless conductivity detection for the analysis of tobramycin and its related substances. J. Pharmaceut. Biomed..

[47] Chen L., Mei M., Huang X. (2017). Development of multiple monolithic fiber solid-phase microextraction and liquid chromatography-tandem mass spectrometry method for the sensitive monitoring of aminoglycosides in honey and milk samples. J. Sep. Sci..

[48] Wang J., Zhao Q., Jiang N., Li W., Chen L., Lin X., Xie Z., You L., Zhang Q. (2017). Urea-formaldehyde monolithic column for hydrophilic in-tube solid-phase microextraction of aminoglycosides. J. Chromatogr. A.

[49] Lehotay S.J., Lightfield A.R. (2018). Simultaneous analysis of aminoglycosides with many other classes of drug residues in bovine tissues by ultrahigh-performance liquid chromatography-tandem mass spectrometry using an ion-pairing reagent added to final extracts. Anal. Bioanal. Chem..

[50] Feng J., She X., He X., Zhu J., Li Y., Deng C. (2018). Synthesis of magnetic graphene/mesoporous silica composites with boronic acid-functionalized pore-walls for selective and efficient residue analysis of aminoglycosides in milk. Food Chem..

[51] Zhang L., Zhu C., Chen C., Zhu S., Zhou J., Wang M., Shang P. (2018). Determination of kanamycin using a molecularly imprinted SPR sensor. Food Chem..

[52] Asakawa D., Uemura M., Sakiyama T., Yamano T. (2018). Sensitivity enhancement of aminoglycosides in hydrophilic interaction liquid chromatography with tandem mass spectrometry by post-column addition of trace sodium acetate in methanol. Food Addit. Contam. Part A Chem. Anal. Control Expo. Risk Assess..

[53] Xu X., Liu Z., Zhao X., Su R., Zhang Y., Shi J., Zhao Y., Wu L., Ma Q., Zhou X. (2013). Ionic liquid-based microwave-assisted surfactant-improved dispersive liquid–liquid microextraction and derivatization of aminoglycosides in milk samples. J. Sep. Sci..

[54] Gupta V.K., Yola M.L., Özaltın N., Atar N., Üstündağ Z., Uzun L. (2013). Molecular imprinted polypyrrole modified glassy carbon electrode for the determination of tobramycin. Electrochim. Acta.

[55] Cai J., Liu W., Gu X. (2004). Microbiological detection of streptomycin residues in milk. Chin. J. Vet. Med..

[56] Wu Q., Peng D., Liu Q., Shabbir M.A.B., Sajid A., Liu Z., Wang Y., Yuan Z. (2019). A novel microbiological method in microtiter plates for screening seven kinds of widely used antibiotics residues in milk, chicken egg and honey. Front. Microbiol..

[57] Gaudin V., Rault A., Hedou C., Soumet C., Verdon E. (2017). Strategies for the screening of antibiotic residues in eggs: Comparison of the validation of the classical microbiological method with an immunobiosensor method. Food Addit. Contam. Part A Chem. Anal. Control Expo. Risk Assess..

[58] Piech T., Majer-Dziedzic B., Kostruba A., Grzelak E.M., Choma I.M. (2016). Thin-layer chromatography-direct bioautography as an alternative method for screening of antibiotic residues in milk: A comparative study. J. Liq. Chromatogr. Relat. Technol..

[59] Zhang X., Wang J., Wu Q., Li L., Wang Y., Yang H. (2019). Determination of kanamycin by high performance liquid chromatography. Molecules.

[60] Yu Y., Liu Y., Wang W., Jia Y., Zhao G., Zhang X., Chen H., Zhou Y. (2019). Highly sensitive determination of aminoglycoside residues in food by sheathless CE-ESI-MS/MS. Anal. Methods.

[61] Kargin I., Sokolova L., Pirogov A., Shpigun O. (2016). HPLC determination of tetracycline antibiotics in milk with post-column derivatization and fluorescence detection. Inorg. Mater..

[62] Zhang X., Wu D., Zhou X., Yu Y., Liu J., Hu N., Wang H., Li G., Wu Y. (2019). Recent progress in the construction of nanozyme-based biosensors and their applications to food safety assay. Trends Analyt. Chem..

[63] Fujii Y., Kaga T., Nishimura K. (2019). Simultaneous determination of aminoglycoside residues in livestock and fishery products by phenylboronic acid solid-phase extraction and liquid chromatography-tandem mass spectrometry. Anal. Sci..

[64] Jariwala F.B., Hibbs J.A., Zhuk I., Sukhishvili S.A., Attygalle A.B. (2020). Rapid determination of aminoglycosides in pharmaceutical preparations by electrospray ionization mass spectrometry. J. Anal. Sci. Technol..

[65] Mehlhorn A., Rahimi P., Joseph Y. (2018). Aptamer-based biosensors for antibiotic detection: A review. Biosensors.

[66] Shalev M., Kandasamy J., Skalka N., Belakhov V., Rosin-Arbesfeld R., Baasov T. (2013). Development of generic immunoassay for the detection of a series of aminoglycosides with 6′-OH group for the treatment of genetic diseases in biological samples. J. Pharm. Biomed. Anal..

[67] Yola M.L., Uzun L., Özaltın N., Denizli A. (2014). Development of molecular imprinted nanosensor for determination of tobramycin in pharmaceuticals and foods. Talanta.

[68] Wu J.X., Zhang S.E., Zhou X.P. (2010). Monoclonal antibody-based ELISA and colloidal gold-based immunochromatographic assay for streptomycin residue detection in milk and swine urine. J. Zhejiang Univ. Sci. B.

[69] Xi X., Zhang M., Li M., Gong Y., Wang M., Chen Z., Wang W. (2013). Development of dcELISA method for rapid detection of streptomycin residue in milk and honey. J. Chin. Inst. Food Sci. Technol..

[70] Wei D., Meng H., Zeng K., Huang Z. (2019). Visual dual dot immunoassay for the simultaneous detection of kanamycin and streptomycin in milk. Anal. Methods.

[71] He J., Wang Y., Zhang X. (2016). Preparation of artificial antigen and development of IgY-based indirect competitive ELISA for the detection of kanamycin residues. Food Anal. Methods.

[72] Xu N., Qu C., Ma W., Xu L., Xu L., Liu L., Kuang H., Xu C. (2011). Development and application of one-step ELISA for the detection of neomycin in milk. Food Agric. Immunol..

[73] Li C., Zhang Y., Eremin S.A., Yakup O., Yao G., Zhang X. (2017). Detection of kanamycin and gentamicin residues in animal-derived food using IgY antibody based ic-ELISA and FPIA. Food Chem..

[74] Verheijen R., Osswald I., Dietrich R., Haasnoot W. (2000). Development of a one step strip test for the detection of (dihydro) streptomycin residues in raw milk. Food Agric. Immunol..

[75] Hendrickson O.D., Byzova N.A., Zvereva E.A., Zherdev A.V., Dzantiev B.B. (2021). Sensitive lateral flow immunoassay of an antibiotic neomycin in foodstuffs. J. Food Sci. Technol..

[76] Sun Y., Xie J., Peng T., Wang J., Xie S., Yao K., Wang C., Sun S., Xia X., Jiang H. (2017). A new method based on time-resolved fluoroimmunoassay for the detection of streptomycin in milk. Food Anal. Methods.

[77] Tao X., Wang J., Xie Y., Zuo X., Mo F., Zhou S., Li H. (2017). Dual-label chemiluminescence strategy for multiplexed immunoassay of 20 fluoroquinolones, 15 β-Lactams, 15 sulfonamides, and cap in milk. Food Anal. Methods.

[78] Zeng H., Chen J., Zhang C., Huang X.-A., Sun Y., Xu Z., Lei H. (2016). Broad-specificity chemiluminescence enzyme immunoassay for (fluoro) quinolones: Hapten design and molecular modeling study of antibody recognition. Anal. Chem..

[79] Yu F., Yu S., Yu L., Li Y., Wu Y., Zhang H., Qu L., Harrington P.d.B. (2014). Determination of residual enrofloxacin in food samples by a sensitive method of chemiluminescence enzyme immunoassay. Food Chem..

[80] Jiang W., Beier R.C., Luo P., Zhai P., Wu N., Lin G., Wang X., Xu G. (2016). Analysis of pirlimycin residues in beef muscle, milk, and honey by a biotin–streptavidin-amplified enzyme-linked immunosorbent assay. J. Agric. Food Chem..

[81] Jin Y., Jang J.W., Lee M.H., Han C.H. (2006). Development of ELISA and immunochromatographic assay for the detection of neomycin. Clin. Chim. Acta.

[82] Wang J., Zhang H., Sheng W., Liu W., Zheng L., Zhang X., Wang S. (2013). Determination of streptomycin residues in animal-derived foods by a reliable and accurate enzyme-linked immunosorbent assay. Anal. Methods.

[83] Abuknesha R.A., Luk C. (2005). Enzyme immunoassays for the analysis of streptomycin in milk, serum and water: Development and assessment of a polyclonal antiserum and assay procedures using novel streptomycin derivatives. Analyst.

[84] Chen Y., Shang Y., Li X., Wu X., Xiao X. (2008). Development of an enzyme-linked immunoassay for the detection of gentamicin in swine tissues. Food Chem..

[85] Jiang L., Wei D., Zeng K., Shao J., Zhu F., Du D. (2018). An enhanced direct competitive immunoassay for the detection of kanamycin and tobramycin in milk using multienzyme-particle amplification. Food Anal. Methods.

[86] Zeng K., Chen B., Li Y., Meng H., Wu Q., Yang J., Liang H. (2022). Gold nanoparticle-carbon nanotube nanohybrids with peroxidase-like activity for the highly-sensitive immunoassay of kanamycin in milk. Int. J. Food Sci. Technol..

[87] Isanga J., Mukunzi D., Chen Y., Suryoprabowo S., Liu L., Kuang H. (2017). Development of a monoclonal antibody assay and immunochromatographic test strip for the detection of amikacin residues in milk and eggs. Food Agric. Immunol..

[88] He J., Hu J., Thirumalai D., Schade R., Du E., Zhang X. (2016). Development of indirect competitive ELISA using egg yolk-derived immunoglobulin (IgY) for the detection of Gentamicin residues. J. Environ. Sci..

[89] Isanga J., Tochi B.N., Mukunzi D., Chen Y., Liu L., Kuang H., Xu C. (2017). Development of a specific monoclonal antibody assay and a rapid testing strip for the detection of apramycin residues in food samples. Food Agric. Immunol..

[90] Sheng W., Yang L., Wang J., Zhang Y., Wang S. (2013). Development of an enzyme-linked immunosorbent assay for the detection of gentamycin residues in animal-derived foods. LWT-Food Sci. Technol..

[91] Chen Y., Chen Q., He L., Shang B., Zhang L. (2012). Enzyme immunoassay and liquid chromatography-fluorescence detection for amikacin in raw milk. Food Chem..

[92] Jin Y., Jang J.W., Han C.H., Lee M.-H. (2006). Development of immunoassays for the detection of kanamycin in veterinary fields. J. Vet. Med. Sci..

[93] Jin Y., Jang J.W., Han C.H., Lee M.H. (2005). Development of ELISA and immunochromatographic assay for the detection of gentamicin. J. Agric. Food Chem..

[94] Byzova N., Zvereva E., Zherdev A., Eremin S., Sveshnikov P., Dzantiev B. (2011). Pretreatment-free immunochromatographic assay for the detection of streptomycin and its application to the control of milk and dairy products. Anal. Chim. Acta.

[95] Pang Y., Zhao S., Liu Z., Chen J., Yang Z., He Z., Shen X., Lei H., Li X. (2022). An enhanced immunochromatography assay based on colloidal gold-decorated polydopamine for rapid and sensitive determination of gentamicin in animal-derived food. Food Chem..

[96] Zhou J., Nie W., Chen Y., Yang C., Gong L., Zhang C., Chen Q., He L., Feng X. (2018). Quadruplex gold immunochromatogaraphic assay for four families of antibiotic residues in milk. Food Chem..

[97] Chen W., Jie W., Chen Z., Jie X., Huang-Xian J. (2012). Chemiluminescent immunoassay and its applications. Chin. J. Anal. Chem..

[98] Gu H., Liu L., Song S., Kuang H., Xu C. (2016). Development of an immunochromatographic strip assay for ractopamine detection using an ultrasensitive monoclonal antibody. Food Agric. Immunol..

[99] Kong D., Xie Z., Liu L., Song S., Kuang H., Xu C. (2017). Development of ic-ELISA and lateral-flow immunochromatographic assay strip for the detection of vancomycin in raw milk and animal feed. Food Agric. Immunol..

[100] Zeng K., Zhang X., Wei D., Huang Z., Cheng S., Chen J. (2020). Chemiluminescence imaging immunoassay for multiple aminoglycoside antibiotics in cow milk. Int. J. Food Sci. Technol..

[101] Zeng K., Zhang Y., Meng H., Chen B., Wu Q., Yang J., Gu X. (2022). Chemiluminescence microarray immunoassay for multiple aminoglycoside antibiotics based on carbon nanotube-assisted signal amplification. Anal. Bioanal. Chem..

[102] Luo P.J., Zhang J.B., Wang H.L., Xia C., Nan W., Zhao Y.F., Wang X.M., Zhang H., Zhang J.Y., Lei Z. (2016). Rapid and sensitive chemiluminescent enzyme immunoassay for the determination of neomycin residues in milk. Biomed. Environ. Sci..

[103] Li Y., Zhang Y., Cao X., Wang Z., Shen J., Zhang S. (2012). Development of a chemiluminescent competitive indirect ELISA method procedure for the determination of gentamicin in milk. Anal. Methods.

[104] Reder-Christ K., Bendas G. (2011). Biosensor applications in the field of antibiotic research-A review of recent developments. Sensors.

[105] Luppa P.B., Sokoll L.J., Chan D.W. (2001). Immunosensors-principles and applications to clinical chemistry. Clin. Chim. Acta.

[106] Wan Y., Su Y., Zhu X., Liu G., Fan C. (2013). Development of electrochemical immunosensors towards point of care diagnostics. Biosens. Bioelectron..

[107] Holford T.R., Davis F., Higson S.P. (2012). Recent trends in antibody based sensors. Biosens. Bioelectron..

[108] Patris S., Vandeput M., Kauffmann J.M. (2016). Antibodies as target for affinity biosensors. Trends Analyt. Chem..

[109] Pollap A., Kochana J. (2019). Electrochemical immunosensors for antibiotic detection. Biosensors.

[110] Wang M., Hu M., Liu J., Guo C., Peng D., Jia Q., He L., Zhang Z., Du M. (2019). Covalent organic framework-based electrochemical aptasensors for the ultrasensitive detection of antibiotics. Biosens. Bioelectron..

[111] Li F., Yu Z., Han X., Lai R.Y. (2019). Electrochemical aptamer-based sensors for food and water analysis: A review. Anal. Chim. Acta.

[112] Gaudin V. (2017). Advances in biosensor development for the screening of antibiotic residues in food products of animal origin-A comprehensive review. Biosens. Bioelectron..

[113] Pan M., Li S., Wang J., Sheng W., Wang S. (2017). Development and validation of a reproducible and label-free surface plasmon resonance immunosensor for enrofloxacin detection in animal-derived foods. Sensors.

[114] Zeng K., Wei W., Jiang L., Zhu F., Du D. (2016). Use of carbon nanotubes as a solid support to establish quantitative (centrifugation) and qualitative (filtration) immunoassays to detect gentamicin contamination in commercial milk. J. Agric. Food Chem..

[115] Zhang Y.F., Gao Z.X. (2017). Antibody development and immunoassays for polycyclic aromatic hydrocarbons (PAHs). Curr. Org. Chem..

[116] Wang H., Sun Y., Li H., Yue W., Kang Q., Shen D. (2018). A smartphone-based ratiometric resonance light scattering device for field analysis of Pb^2+^ in river water samples and immunoassay of alpha fetoprotein using PbS nanoparticles as signal tag. Sens. Actuators B Chem..

[117] Fruhmann P., Sanchis A., Mayerhuber L., Vanka T., Kleber C., Salvador J.P., Marco M.P. (2018). Immunoassay and amperometric biosensor approaches for the detection of deltamethrin in seawater. Anal. Bioanal. Chem..

[118] Felix F.S., Angnes L. (2018). Electrochemical immunosensors-a powerful tool for analytical applications. Biosens. Bioelectron..

[119] AlRabiah H., Hamidaddin M.A., Darwish I.A. (2019). Automated flow fluorescent noncompetitive immunoassay for measurement of human plasma levels of monoclonal antibodies used for immunotherapy of cancers with KinExA™ 3200 biosensor. Talanta.

[120] Chiu N.F., Lin T.L., Kuo C.T. (2018). Highly sensitive carboxyl-graphene oxide-based surface plasmon resonance immunosensor for the detection of lung cancer for cytokeratin 19 biomarker in human plasma. Sens. Actuators B Chem..

[121] Ou Y., Jin X., Fang J., Tian Y., Zhou N. (2020). Multi-cycle signal-amplified colorimetric detection of tobramycin based on dual-strand displacement and three-way DNA junction. Microchem. J..

[122] Mishra G.K., Sharma A., Bhand S. (2015). Ultrasensitive detection of streptomycin using flow injection analysis-electrochemical quartz crystal nanobalance (FIA-EQCN) biosensor. Biosens. Bioelectron..

[123] De-los-Santos-Álvarez N., Lobo-Castañón M.J., Miranda-Ordieres A.J., Tuñón-Blanco P. (2009). SPR sensing of small molecules with modified RNA aptamers: Detection of neomycin B. Biosens. Bioelectron..

[124] Kokkinos C., Economou A., Prodromidis M.I. (2016). Electrochemical immunosensors: Critical survey of different architectures and transduction strategies. Trends Analyt. Chem..

[125] Xu Y., Han T., Li X., Sun L., Zhang Y., Zhang Y. (2015). Colorimetric detection of kanamycin based on analyte-protected silver nanoparticles and aptamer-selective sensing mechanism. Anal. Chim. Acta.

[126] Yin J., Guo W., Qin X., Pei M., Wang L., Ding F. (2016). A regular “signal attenuation” electrochemical aptasensor for highly sensitive detection of streptomycin. New J. Chem..

[127] Yan Q., Cao L., Dong H., Tan Z., Hu Y., Liu Q., Liu H., Zhao P., Chen L., Liu Y. (2019). Label-free immunosensors based on a novel multi-amplification signal strategy of TiO_2_-NGO/Au@Pd hetero-nanostructures. Biosens. Bioelectron..

[128] Wu M.F., Wang Y., Li S., Dong X.X., Yang J.Y., Shen Y.D., Wang H., Sun Y.M., Lei H.T., Xu Z.L. (2019). Ultrasensitive immunosensor for acrylamide based on chitosan/SnO_2_-SiC hollow sphere nanochains/gold nanomaterial as signal amplification. Anal. Chim. Acta.

[129] Zhang C., Zhang S., Jia Y., Li Y., Wang P., Liu Q., Xu Z., Li X., Dong Y. (2019). Sandwich-type electrochemical immunosensor for sensitive detection of CEA based on the enhanced effects of Ag NPs@ CS spaced Hemin/rGO. Biosens. Bioelectron..

[130] Supraja P., Sudarshan V., Tripathy S., Agrawal A., Singh S.G. (2019). Label free electrochemical detection of cardiac biomarker troponin T using ZnSnO_3_ perovskite nanomaterials. Anal. Methods.

[131] Supraja P., Tripathy S., Vanjari S.R.K., Singh V., Singh S.G. (2019). Label free, electrochemical detection of atrazine using electrospun Mn2O3 nanofibers: Towards ultrasensitive small molecule detection. Sens. Actuators B Chem..

[132] Wei Q., Zhao Y., Du B., Wu D., Li H., Yang M. (2012). Ultrasensitive detection of kanamycin in animal derived foods by label-free electrochemical immunosensor. Food. Chem..

[133] Chen Y.P., Zou M., Qi C., Xie M.X., Wang D.N., Wang Y.F., Xue Q., Li J.F., Chen Y. (2013). Immunosensor based on magnetic relaxation switch and biotin-streptavidin system for the detection of kanamycin in milk. Biosens. Bioelectron..

[134] Li F., Yu Z., Han X., Shi W., Liu Y., Yan H., Zhang G. (2018). A signal-on electrochemical aptasensor for highly sensitive and specific detection of kanamycin based on target-induced signaling probe shifting mechanism. Sens. Actuators B Chem..

[135] Sharma A., Istamboulie G., Hayat A., Catanante G., Bhand S., Marty J.L. (2017). Disposable and portable aptamer functionalized impedimetric sensor for detection of kanamycin residue in milk sample. Sens. Actuators B Chem..

[136] Taranova N., Berlina A., Zherdev A., Dzantiev B. (2015). ‘Traffic light’immunochromatographic test based on multicolor quantum dots for the simultaneous detection of several antibiotics in milk. Biosens. Bioelectron..

[137] Wang P., Wang R., Zhang W., Su X., Luo H. (2016). Novel fabrication of immunochromatographic assay based on up conversion phosphors for sensitive detection of clenbuterol. Biosens. Bioelectron..

[138] Zhang Z., Lin M., Zhang S., Vardhanabhuti B. (2013). Detection of aflatoxin M1 in milk by dynamic light scattering coupled with superparamagnetic beads and gold nanoprobes. J. Agric. Food Chem..

[139] Song E., Yu M., Wang Y., Hu W., Cheng D., Swihart M.T., Song Y. (2015). Multi-color quantum dot-based fluorescence immunoassay array for simultaneous visual detection of multiple antibiotic residues in milk. Biosens. Bioelectron..

[140] Bunzli J.C.G. (2010). Lanthanide luminescence for biomedical analyses and imaging. Chem. Rev..

[141] Hagan A., Zuchner T. (2011). Lanthanide-based time-resolved luminescence immunoassays. Anal. Bioanal. Chem..

[142] Wang Z., Sun Y., Liang D., Zeng Y., He S., Mari G.M., Peng T., Jiang H. (2020). Highly sensitive chromatographic time-resolved fluoroimmunoassay for rapid onsite detection of streptomycin in milk. J. Dairy Sci..

[143] Wang Y.-F., Wang D.-N., Zou M.-Q., Jin Y., Yun C.L., Gao X.W. (2011). Application of suspension array for simultaneous detection of antibiotic residues in raw milk. Anal. Lett..

[144] Tamayo F., Turiel E., Martín-Esteban A. (2007). Molecularly imprinted polymers for solid-phase extraction and solid-phase microextraction: Recent developments and future trends. J. Chromatogr. A.

[145] Lv Y., Tan T., Svec F. (2013). Molecular imprinting of proteins in polymers attached to the surface of nanomaterials for selective recognition of biomacromolecules. Adv. Biochem. Eng..

[146] Ren Y., Ma W., Ma J., Wen Q., Wang J., Zhao F. (2012). Synthesis and properties of bisphenol A molecular imprinted particle for selective recognition of BPA from water. J. Colloid Interface Sci..

[147] Tang S.P., Canfarotta F., Smolinska-Kempisty K., Piletska E., Guerreiro A., Piletsky S. (2017). A pseudo-ELISA based on molecularly imprinted nanoparticles for detection of gentamicin in real samples. Anal. Methods.

[148] Que X., Liu B., Fu L., Zhuang J., Chen G., Tang D. (2013). Molecular imprint for electrochemical detection of streptomycin residues using enzyme signal amplification. Electroanalysis.

[149] Liu B., Tang D., Zhang B., Que X., Yang H., Chen G. (2013). Au (III)-promoted magnetic molecularly imprinted polymer nanospheres for electrochemical determination of streptomycin residues in food. Biosens. Bioelectron..

[150] Zhang M., Zhang B., Li T., Zhu X., Guo W. (2022). Electrochemical detection of aminoglycoside antibiotics residuals in milk based on magnetic molecularly imprinted particles and metal ions. Food Chem..

[151] Frasconi M., Tel-Vered R., Riskin M., Willner I. (2010). Surface plasmon resonance analysis of antibiotics using imprinted boronic acid-functionalized Au nanoparticle composites. Anal. Chem..

[152] Han S., Li B., Song Z., Pan S., Zhang Z., Yao H., Zhu S., Xu G. (2017). A kanamycin sensor based on an electrosynthesized molecularly imprinted poly-o-phenylenediamine film on a single-walled carbon nanohorn modified glassy carbon electrode. Analyst.

[153] Yu C., Li N., Zhang R., Xie D., Li F., Cao Q. (2022). Reduced graphene oxide/poly (2-Aminopyridine) modified molecularly imprinted glassy carbon electrode (GCE) for the determination of kanamycin in milk and pork by differential pulse voltammetry (DPV). Anal. Lett..

[154] Chen Y., Wang Y., Liu L., Wu X., Xu L., Kuang H., Li A., Xu C. (2015). A gold immunochromatographic assay for the rapid and simultaneous detection of fifteen β-lactams. Nanoscale.

[155] Zhang X., Yu X., Wen K., Li C., Mujtaba Mari G., Jiang H., Shi W., Shen J., Wang Z. (2017). Multiplex lateral flow immunoassays based on amorphous carbon nanoparticles for detecting three fusarium mycotoxins in maize. J. Agric. Food Chem..

[156] Liu J., Zeng J., Tian Y., Zhou N. (2018). An aptamer and functionalized nanoparticle-based strip biosensor for on-site detection of kanamycin in food samples. Analyst.

[157] Wei D., Zhang X., Chen B., Zeng K. (2020). Using bimetallic Au@Pt nanozymes as a visual tag and as an enzyme mimic in enhanced sensitive lateral-flow immunoassays: Application for the detection of streptomycin. Anal. Chim. Acta.

[158] Jiang J., Luo P., Liang J., Shen X., Lei H., Li X. (2022). A highly sensitive and quantitative time resolved fluorescent microspheres lateral flow immunoassay for streptomycin and dihydrostreptomycin in milk, honey, muscle, liver, and kidney. Anal. Chim. Acta.

[159] Liu C., Jiang Y., Xiu L., Qian R., Zhao M., Luo P., Ke Y., Li G., Jiang W. (2021). Ultratrace analysis of neomycin residues in milk at femtogram levels by flow-through immunoaffinity chromatography test. Food Anal. Methods.

[160] Xiao X., Hu S., Lai X., Peng J., Lai W. (2021). Developmental trend of immunoassays for monitoring hazards in food samples: A review. Trends Food Sci. Technol..

[161] Zhang J., Wang Y., Lu X. (2021). Molecular imprinting technology for sensing foodborne pathogenic bacteria. Anal. Bioanal. Chem..

[162] Jia M., E Z., Zhai F., Bing X. (2020). Rapid multi-residue detection methods for pesticides and veterinary drugs. Molecules.

[163] Zhao Q., Lu D., Zhang G., Zhang D., Shi X. (2021). Recent improvements in enzyme-linked immunosorbent assays based on nanomaterials. Talanta.

[164] He H., Liu B., Wen S., Liao J., Lin G., Zhou J., Jin D. (2018). Quantitative lateral flow strip sensor using highly doped upconversion nanoparticles. Anal. Chem..

